# Longitudinal clinical and functional outcome in distinct cognitive subgroups of first-episode psychosis: a cluster analysis

**DOI:** 10.1017/S0033291721004153

**Published:** 2023-04

**Authors:** Priscilla P. Oomen, Marieke J. H. Begemann, Bodyl A. Brand, Lieuwe de Haan, Wim Veling, Sanne Koops, Jim van Os, Filip Smit, P. Roberto Bakker, Nico van Beveren, Nynke Boonstra, Sinan Gülöksüz, Martijn Kikkert, Joran Lokkerbol, Machteld Marcelis, Bram-Sieben Rosema, Franciska de Beer, Shiral S. Gangadin, Chris N. W. Geraets, Erna van ‘t Hag, Yudith Haveman, Inge van der Heijden, Alban E. Voppel, Elske Willemse, Therese van Amelsvoort, Maarten Bak, Albert Batalla, Agaath Been, Marinte van den Bosch, Truus van den Brink, Gunnar Faber, Koen P. Grootens, Martin de Jonge, Rikus Knegtering, Jörg Kurkamp, Amrita Mahabir, Gerdina H. M. Pijnenborg, Tonnie Staring, Natalie Veen, Selene Veerman, Sybren Wiersma, Ellen Graveland, Joelle Hoornaar, Iris E. C. Sommer

**Affiliations:** 1 Department of Biomedical Sciences of Cells and Systems, and Department of Psychiatry, University of Groningen, University Medical Center Groningen, Groningen, The Netherlands; 2 Department of Early Psychosis, Amsterdam UMC, Academic Medical Center, Amsterdam, The Netherlands; 3 Department of Psychiatry, University of Groningen, University Medical Center Groningen, Groningen, The Netherlands; 4 Department of Psychiatry, UMC Utrecht Brain Center, University Medical Center Utrecht, Utrecht, The Netherlands; 5 Department of Psychiatry and Neuropsychology, School for Mental Health and Neuroscience (MheNS), Maastricht University Medical Centre, Maastricht, The Netherlands; 6 King's College London, King's Health Partners Department of Psychosis Studies, Institute of Psychiatry, Psychology & Neuroscience, London, UK; 7 Department of Epidemiology and Biostatistics, Amsterdam Public Health Research Institute, Amsterdam University Medical Centers, location VUmc, Amsterdam, The Netherlands; 8 Department of Clinical, Neuro and Developmental Psychology, Amsterdam Public Health Research Institute, Vrije Universiteit, Amsterdam, The Netherlands; 9 Centre of Economic Evaluation & Machine Learning, Trimbos Institute (Netherlands Institute of Mental Health), Utrecht, The Netherlands; 10 Department of Research, Arkin Mental Health Care, Amsterdam, The Netherlands; 11 Antes Center for Mental Health Care, Rotterdam, The Netherlands; 12 Department of Neuroscience, Erasmus MC, Rotterdam, The Netherlands; 13 Department of Psychiatry, Erasmus MC, Rotterdam, The Netherlands; 14 NHL/Stenden, University of Applied Sciences, Leeuwarden, The Netherlands; 15 KieN VIP Mental Health Care Services, Leeuwarden, The Netherlands; 16 Department of Psychiatry, Yale University School of Medicine, New Haven, Connecticut, USA; 17 Department of Psychiatry and Neuropsychology, School for Mental Health and Neuroscience, EURON, Maastricht University Medical Center, Maastricht, The Netherlands; 18 Institute for Mental Health Care Eindhoven (GGzE), Eindhoven, The Netherlands; 19 Janssen-Cilag B.V., Breda, the Netherlands; 20 Mondriaan Mental Health Care, Heerlen, The Netherlands; 21 Dimence Institute for Mental Health, Deventer, Zwolle, The Netherlands; 22 Early Intervention Team, GGZ Centraal, Amersfoort, The Netherlands; 23 Yulius, Mental Health Institute, Dordrecht, The Netherlands; 24 Reinier van Arkel Institute for Mental Health Care, ‘s Hertogenbosch, The Netherlands; 25 Program for Psychosis & Severe Mental Illness, Pro Persona Mental Health, Wolfheze, The Netherlands; 26 Lentis Research, Lentis Psychiatric Institute, Groningen, The Netherlands; 27 Center for Youth with Psychosis, Mediant ABC Twente, Enschede, The Netherlands; 28 Early Psychosis Team, GGNet, Apeldoorn, The Netherlands; 29 Department of Psychotic Disorders, GGZ-Drenthe, Assen, The Netherlands; 30 Department of Clinical and Developmental Neuropsychology, Faculty BSS, University of Groningen, Groningen, The Netherlands; 31 Department ABC Early Psychosis, Altrecht Psychiatric Institute, Utrecht, The Netherlands; 32 GGZ Delfland, Delfland Institute for Mental Health Care, Delft, The Netherlands; 33 Community Mental Health, Mental Health Service Noord-Holland Noord, Alkmaar, The Netherlands; 34 Early Intervention Psychosis Team, GGZ inGeest Specialized Mental Health Care, Hoofddorp, The Netherlands

**Keywords:** Clustering, cognition, FEP, functional outcome, global functioning, psychosis

## Abstract

**Background:**

Cognitive deficits may be characteristic for only a subgroup of first-episode psychosis (FEP) and the link with clinical and functional outcomes is less profound than previously thought. This study aimed to identify cognitive subgroups in a large sample of FEP using a clustering approach with healthy controls as a reference group, subsequently linking cognitive subgroups to clinical and functional outcomes.

**Methods:**

204 FEP patients were included. Hierarchical cluster analysis was performed using baseline brief assessment of cognition in schizophrenia (BACS). Cognitive subgroups were compared to 40 controls and linked to longitudinal clinical and functional outcomes (PANSS, GAF, self-reported WHODAS 2.0) up to 12-month follow-up.

**Results:**

Three distinct cognitive clusters emerged: relative to controls, we found one cluster with preserved cognition (*n* = 76), one moderately impaired cluster (*n* = 74) and one severely impaired cluster (*n* = 54). Patients with severely impaired cognition had more severe clinical symptoms at baseline, 6- and 12-month follow-up as compared to patients with preserved cognition. General functioning (GAF) in the severely impaired cluster was significantly lower than in those with preserved cognition at baseline and showed trend-level effects at 6- and 12-month follow-up. No significant differences in self-reported functional outcome (WHODAS 2.0) were present.

**Conclusions:**

Current results demonstrate the existence of three distinct cognitive subgroups, corresponding with clinical outcome at baseline, 6- and 12-month follow-up. Importantly, the cognitively preserved subgroup was larger than the severely impaired group. Early identification of discrete cognitive profiles can offer valuable information about the clinical outcome but may not be relevant in predicting self-reported functional outcomes.

## Introduction

Despite relatively successful treatment of clinical symptoms after first-episode psychosis (FEP) (Kahn *et al*. 2018), many patients continue to experience ongoing functional impairment in day-to-day life (Henry *et al*. 2010; Lally *et al*. 2017). Large variability exists in the outcome of FEP with recovery rates ranging from 13.5% to 38% (Jääskeläinen *et al*. 2013; Lally *et al*. 2017). A significant minority of patients shows the excellent recovery, but a large proportion of patients continues to exhibit moderate or severe functional impairment (Jääskeläinen *et al*. 2013; Lally *et al*. 2017). Recovery rates appear to be stable 2 years after illness onset as demonstrated in a large meta-analysis (Lally *et al*. 2017), underscoring the importance of identifying factors that can predict outcome overtime in the early stages of disease onset. However, most longitudinal studies have examined predictors at the diagnostic group level and do not take the high heterogeneity between individual patients with the same diagnosis into account (Santesteban-Echarri *et al*. 2017; Suvisaari *et al*. 2018). This hampers a more personalized treatment approach in clinical care, as individuals require treatment tailored to their illness profile.

During past years, cognitive impairment received substantial attention because of its presence prior to illness onset and associations with both clinical and functional outcomes over time (Helldin, Mohn, Olsson, & Hjärthag, 2020; Johansson, Hjärthag, & Helldin, 2020; Lindgren, Holm, Kieseppä, & Suvisaari, 2020; Santesteban-Echarri *et al*. 2017). Some authors have considered cognitive dysfunction to be the core feature of schizophrenia (Heinrichs, 2005). However, recent literature shows that global cognitive deficits are not a general finding, as it is becoming increasingly apparent that several cognitive subgroups may exist within the FEP population, including a substantial subset of patients that remains cognitively intact (Carruthers, Van Rheenen, Gurvich, Sumner, & Rossell, 2019; Moritz *et al*. 2017; Uren, Cotton, Killackey, Saling, & Allott, 2017). Also, the predictive value of cognitive deficits in terms of functional impairment may be less pronounced as previously thought. Notably, a recent meta-analysis showed only small to medium effect sizes for the association between cognition and functional outcome, leaving a significant proportion of the variance unexplained (Halverson *et al*. 2019). It is plausible that variance in both functional and clinical outcomes may be related to differences in severity of cognitive dysfunction. Indeed, it has been demonstrated that cognitive performance in a “neuropsychologically normal” range does not correlate well with aspects of everyday functioning whereas more severe levels of cognitive impairment do seem to be associated with functional outcomes (Strassnig *et al*. 2018). This underscores the value of grouping FEP patients into subtypes along the cognitive continuum, demonstrating possible subgroups with distinct illness profiles.

An essential and relatively novel solution for determining homogeneous subgroups is a data-driven clustering approach. Defining subgroups based on baseline cognitive profile may provide crucial information regarding functional outcome and prognosis. Such information is urgently needed, as the high heterogeneity and lack of good predictors hamper clinicians in providing optimal care for individual patients. Early identification of risk factors associated with poor outcomes is highly valuable as this would aid individually tailored interventions that may positively impact the long-term outcome.

The current study includes a large sample of FEP patients who were 3–6 months in remission of their psychotic symptoms at baseline, to identify homogeneous subgroups of cognition based on a data-driven clustering approach. Factors that may influence cognitive function, such as the distraction by unusual ideas and/or hallucinations, long-term antipsychotic medication use or the duration of illness, are limited in the current sample as all patients were in a similar early stage of their illness. Emergent cognitive subgroups were subsequently compared to healthy controls to assess the level of cognitive (under)performance. Cognitive subgroups were then evaluated regarding clinical [Positive and Negative Syndrome Scale (PANSS), Global Assessment of Functioning (GAF)] and functional [WHO Disability Assessment Scale 2.0 (WHODAS2.0)] outcome at baseline and longitudinally at 6- and 12-month follow-up. The clinician-rated GAF has been widely used in clinical and research settings and has been adopted as meaningful, however, the DSM-5 recommends a new tool for the assessment of global functioning and impairment, the WHODAS 2.0, a patient self-report assessment tool that evaluates the patient's ability to perform activities in six domains of functioning (Gold, 2014). Based on a recent systematic review regarding cognitive subgrouping studies in schizophrenia spectrum disorders, we expected to find three distinct cognitive subtypes; a relatively intact cognitive subgroup, an intermediate cognitive subgroup and a globally impaired subgroup (Carruthers *et al*. 2019). We further hypothesized that emergent cognitive subtypes are characterized by differences in both clinical and functional outcomes at baseline and follow-up.

## Method

### Participants

Data were used from the ongoing Handling Antipsychotic Medication: Long-term Evaluation of Targeted Treatment (HAMLETT) study (Begemann *et al*. 2020). Patients were recruited from outpatient settings in 24 healthcare centers throughout the Netherlands. Written informed consent was obtained from all participants and study procedures were performed according to the Declaration of Helsinki (64^th^ WMA general assembly; October 2013). Ethics approval was obtained from the research and ethics committee of the University Medical Center Groningen, the Netherlands (protocol number: NL 62202.042.17, trial registration EudraCT number: 2017-002406-12). Recruitment and study procedures are described in detail by Begemann *et al*. (2020).

In short, the current study included data from 204 patients aged between 16 and 60 years old with the first episode of schizophrenia, schizoaffective disorder, schizophreniform disorder, brief psychotic disorder, delusional disorder, substance/medication-induced psychotic disorder, or those classified as Unspecified Schizophrenia Spectrum and Other Psychotic Disorders (DSM-5, or as described in the International Classification of Diseases-10). Diagnosis and duration of illness were established by their treating psychiatrist and confirmed by the Comprehensive Assessment of Symptoms and History (CASH) (Andreasen, Flaum, & Arndt, 1992). At baseline, all patients were 3–6 months in remission of their first psychotic episode and used antipsychotic medication. Symptomatic remission is defined as “sustained improvement of psychotic symptoms to the level that any remaining psychotic symptoms (such as hallucinatory experiences, unusual thought content, conceptual disorganization) are mild, which means (consistent with international remission criteria) that they do not interfere with behavior and daily functioning.”

Self-reports of current antipsychotic medication use (mg/day) were converted into a chlorpromazine equivalent (CPZE, mg/day) for each patient (Gardner, Murphy, O'Donnell, Centorrino, & Baldessarini, 2010). The highest educational level achieved (CASH) (Andreasen *et al*. 1992), was converted into the number of years of education (YOE; see Online Supplementary Table S1).

Moreover, 40 healthy controls were included as a reference group for cognitive functioning. Healthy controls did not have any history of psychiatric illness and were aged between 19 and 45 years (Trial registration: ABR NL50657.041.14).

### Procedures

#### Cognitive testing

Cognitive performance was assessed at baseline using the Dutch version of the brief assessment of cognition in schizophrenia (BACS) (Keefe *et al*. 2004). The test consists of six subtests that assess different cognitive domains, including:

1. List Learning – Verbal memory
2. Digit Sequencing Task – Working memory
3. Token Motor Task – Motor speed
4. Category Instances and Controlled Oral Word Association Test – Verbal fluency
5. Symbol Coding – Attention and information processing speed
6. Tower of London – Executive function

Performances of all participants on the subtests of the BACS were standardized by creating z-scores adjusted for gender and age using the norms of Keefe *et al*. (2004). A composite z-score was calculated by averaging all of the six standardized primary measures from the BACS. Participants missing more than 2 cognitive sub-scores were excluded from analysis (*n* = 2). For participants with ⩽2 missing sub-scores, scores were replaced by the corresponding population mean for that specific domain (*n* = 8).

#### Clinical outcome

Clinical symptomatology was assessed by trained central study personnel using the Positive and Negative Symptom Scale (PANSS) at baseline, 6 months and 12-month follow-up (Kay, Fiszbein, & Opler, 1987).

In addition, clinical global functioning was evaluated by trained central study personnel at baseline, 6 months and 12-month follow-up using the GAF (Jones, Thornicroft, Coffey, & Dunn, 1995).

To ensure data quality, assessors are comprehensively trained and the central team of assessors have biannual meetings during which inter-rater reliability is assessed and protocol adherence is checked.

#### Self-reported functional outcome

Self-reported global functioning and disability were evaluated at baseline, 6 months and 12-month follow-up using the WHO Disability Assessment Schedule 2.0 (WHODAS 2.0). This questionnaire consists of 36 items covering six domains of functioning in everyday life: cognition (understanding and communicating), mobility (moving and getting around), self-care (hygiene, eating, and staying alone), getting along (interacting with other people), life activities (domestic responsibilities, leisure, work and school) and participation (joining in community activities). Participants respond to each item on a 5-point scale from 0 (No Difficulty) to 4 (Extreme Difficulty/Cannot Do). Overall scores range from 0 to 100 with higher scores indicating a greater level of self-reported disability (Üstün, 2010).

### Statistical analyses

Statistical analyses were performed using Statistical Package for Social Sciences (SPSS) version 25 (IBM Corp. Released 2017. IBM SPSS Statistics for Windows, Version 25.0. Armonk, NY: IBM Corp.). Healthy controls and FEP patients were compared on demographic variables such as gender, age, years of education and cognitive performance using Pearson's chi-square (categorical variables) and one-way analysis of variance (ANOVA, continuous variables).

Patients were clustered based on their composite BACS Z-score, as all domains of cognitive functioning that are assessed by the BACS are found to be consistently impaired in schizophrenia (Keefe *et al*. 2004). A hierarchical clustering approach (HCA) was performed for the total sample of patients. Case similarity was computed using squared Euclidean distance and Ward's linkage was used as agglomeration procedure specification. By using Ward's method, the difference or distance between two clusters is defined by the increase of the sum of squares when merging them (Ward, 1963). After careful inspection of the dendrogram and meaningful jumps in the agglomeration schedule coefficients, the optimal number of clusters was defined. For the dendrogram and agglomeration schedule, see Supplementary Figure S1 and S2. Next, a *k*-means clustering technique was applied to optimize the retained clusters. The number of *k* clusters and initial partitions in the *k*-means solution was defined by results obtained from the hierarchical clustering procedure.

Emergent cognitive patient clusters were then compared to a group of healthy controls to verify the level of cognitive (under)performance. Furthermore, differences in demographic variables and clinical characteristics were assessed using Pearson's χ^2^ (categorical variables) and One-way Analysis of Variance (ANOVA, continuous variables). Subsequently, cognitive patient clusters were compared on both clinical (PANSS, GAF) and functional (WHODAS 2.0) outcomes using ANOVA for baseline comparisons and ANCOVA for comparisons at 6- and 12-month follow-up. Post hoc comparisons were conducted for all significant ANOVA and ANCOVA effects, using Bonferroni correction for multiple comparisons.

## Results

### Demographics

A total of 204 patients and 40 healthy controls were included at baseline. Sociodemographic and clinical characteristics are presented in Online Supplementary Table S2. The group of patients consisted of 148 males (72.5%), the healthy controls included 32 males (80.0%). Patients were significantly older (*M* = 27.93, s.d. = 8.90) than healthy individuals (*M* = 24.48, s.d. = 4.98), (*p* = 0.018). Patients attained fewer years of education compared to healthy controls (*p* = 0.006) and patients scored significantly worse on both the BACS composite and all subtests (all *p* < 0.001, executive functioning *p* = 0.047). At 6- and 12-month follow-up, the sample consisted of 145 and 132 patients respectively.

### Cluster solution

Hierarchical clustering (Ward's method) and *K*-means optimization using BACS composite scores for the total sample of patients resulted in three distinct cognitive clusters (Table 1). Subgroups were subsequently compared to a group of healthy controls to assess the level of cognitive (under)performance.

**Table 1.** Mean (s.d.) baseline demographic and cognitive characteristics for FEP cognitive clusters and healthy controls

		FEP patients (*n* = 204)						
	Healthy controls (*n* = 40)	Preserved cognition (*n* = 76)	Moderately impaired cognition (*n* = 74)	Severely impaired cognition (*n* = 54)	Test statistic	df	*p* value	Post hoc analyses*
					*F*, χ^2^			
Male, *n* (%)	32 (80.0%)	47 (61.8%)	60 (81.1%)	41 (75.9%)	χ^2^ = 8.56	3	*p* = 0.036	a, d
Age	24.48 (4.98)	29.62 (10.26)	26.54 (7.26)	27.46 (8.65)	*F* = 3.70	3	*p* = 0.012	a
Years of education	14.95 (1.95)	14.75 (1.93)	13.42 (2.15)	12.84 (3.28)	*F* = 10.31	3	*p* < 0.001	b, c, d, e
Years of education parents	13.63 (2.20)	13.61 (2.68)	12.52 (3.87)	12.15 (3.06)	*F* = 3.11	3	*p* = 0.027	–
Chlorpromazine equivalent	N.A.	210.11 (127.24)	258.62 (139.43)	249.88 (145.86)	*F* = 2.26	2	*p* = 0.107	–
BACS, Z-score								
Composite score	0.13 (1.15)	−0.18 (0.53)	−1.59 (0.39)	−3.00 (0.69)	*F* = 247.51	3	*p* < 0.001	b, c, d, e, f
Verbal memory	0.43 (1.01)	0.05 (0.84)	−0.83 (0.91)	−1.57 (0.85)	*F* = 53.43	3	*p* < 0.001	b, c, d, e, f
Working memory	0.07 (1.05)	−0.13 (0.88)	−0.97 (0.94)	−2.05 (1.00)	*F* = 55.49	3	*p* < 0.001	b, c, d, e, f
Motor speed	−0.15 (0.96)	−0.01 (0.92)	−1.06 (1.18)	−2.09 (1.21)	*F* = 44.93	3	*p* < 0.001	b, c, d, e, f
Verbal fluency	0.11 (1.05)	−0.18 (1.12)	−1.33 (0.80)	−1.91 (0.73)	*F* = 56.24	3	*p* < 0.001	b, c, d, e, f
Attention & Processing speed	−0.23 (1.19)	−0.80 (0.94)	−1.43 (0.66)	−1.89 (0.73)	*F* = 33.91	3	*p* < 0.001	a, b, c, d, e, f
Executive function	0.24 (0.87)	0.49 (0.80)	−0.07 (0.72)	−1.21 (1.61)	*F* = 30.40	3	*p* < 0.001	c, d, e, f

FEP, first-episode psychosis; BACS, brief assessment of cognition in schizophrenia; df, degrees of freedom.

* a HC significantly different from the relatively preserved cluster; b HC significantly different from the moderately impaired cluster; c HC significantly different from the severely impaired cluster; d relatively preserved cluster significantly different from moderately impaired cluster; e relatively preserved cluster significantly different from severely impaired cluster; f moderately impaired cluster significantly different from a severely impaired cluster.

**Fig. 1.**
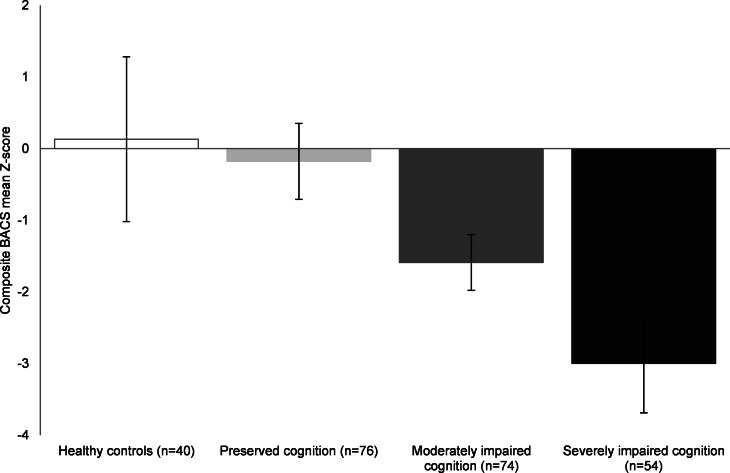
BACS composite means Z-scores illustrated for FEP cognitive clusters and healthy controls Error bars represent standard deviations. All groups showed significant differences (*p* < 0.05) except for healthy controls compared to the preserved cognitive cluster. BACS, brief assessment of cognition in schizophrenia; FEP, first-episode psychosis.

**Fig. 2.**
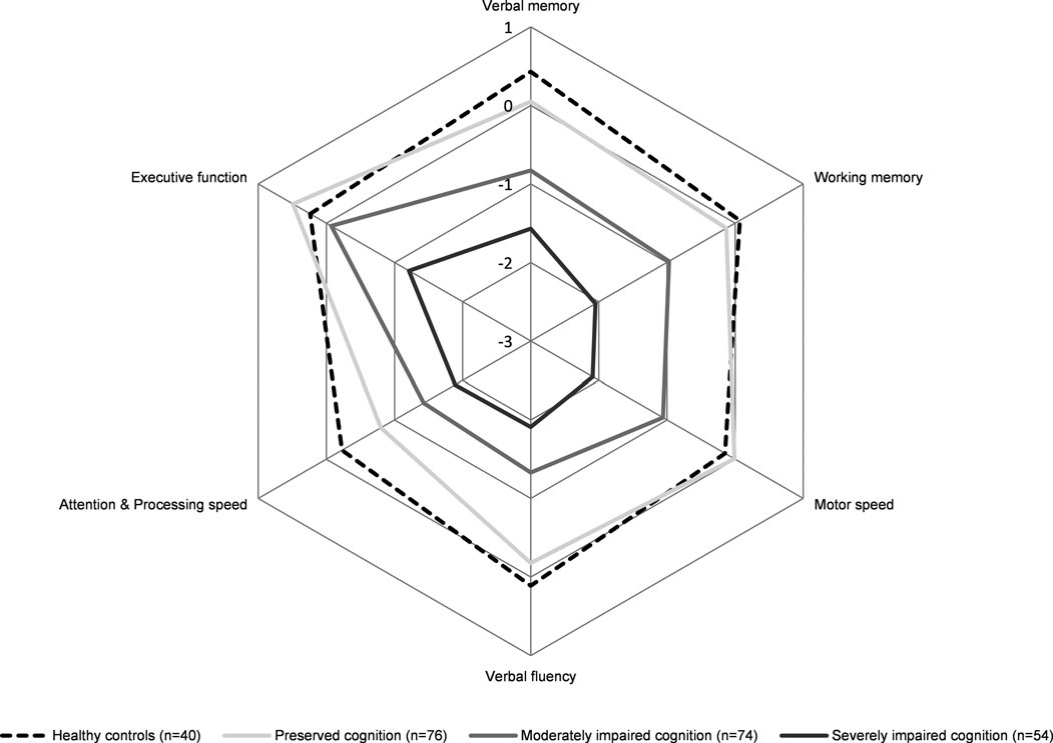
BACS subdomain means Z-scores illustrated for FEP cognitive clusters and healthy controls Pentagons represent mean BACS Z-scores. For detailed statistics, see Table 1. BACS, brief assessment of cognition in schizophrenia; FEP, first-episode psychosis.

One cluster could be described as a relatively preserved group (*n* = 76). The BACS composite score was not significantly different compared to healthy controls, yet these patients scored significantly lower on attention and processing speed compared to healthy controls (*p* = 0.008). An intermediate or moderately impaired cognitive cluster (*n* = 74) displaying reduced functioning on all cognitive domains compared to healthy controls (all *p* < 0.001), except for executive function (*p* = 0.730) was observed. Lastly, the severely impaired cognitive cluster (*n* = 54) showed significant impairments across all domains assessed relative to the controls, with working memory and motor speed showing the most severe deficits (all *p* < 0.001). Results are demonstrated in Figs 1 and 2.

The relatively preserved cluster was significantly older than the healthy controls (*p* = 0.011), but no age differences were demonstrated between the three cognitive patient clusters. The moderately impaired cluster and severely impaired cluster had received significantly fewer years of education compared to both the healthy controls (*p* = 0.007 and *p* < 0.001, respectively) and the relatively preserved cluster (*p* = 0.004 and *p* < 0.001, respectively). Parental years of education attained showed an overall effect (*F*(3) = 3.11, *p* = 0.027) but no significant differences between clusters. Furthermore, chlorpromazine equivalents were not significantly different between clusters (*p* = 0.107).

### Clinical outcome

**Fig. 3.**
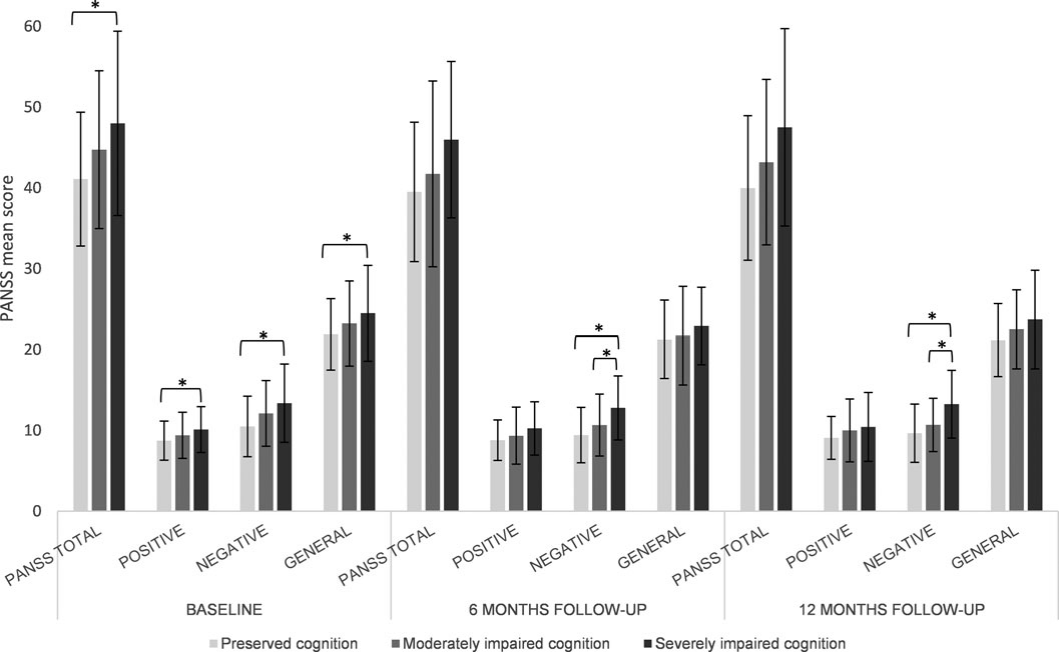
PANSS mean scores illustrated for FEP cognitive clusters at baseline, 6-month follow-up and 12-month follow-up comparisons at 6- and 12-month follow-up were corrected for clinical symptoms at baseline. * illustrates *p* < 0.05; Error bars represent standard deviations. PANSS, Positive and Negative Syndrome Scale; FEP, first-episode psychosis.

Although all patients were in clinical remission at baseline, the subgroup of patients with severely impaired cognition had significantly higher symptom severity compared to the cognitively preserved subgroup, with higher scores on the PANSS total subscale (*p* < 0.001), as well as the positive (*p* = 0.014), negative (*p* < 0.001) and general subscales (*p* = 0.014). Results are demonstrated in Fig. 3.

After correcting for clinical symptoms at baseline, the patient groups with severely impaired and preserved cognitive performance showed significant differences on PANSS negative symptomatology at 6- and 12-month follow-up (*n* = 145, *p* = 0.017; *n* = 132, *p* = 0.018, respectively). Those with severely impaired and moderately impaired cognitive performance differed on the PANSS negative subscale (6 months: *p* = 0.010; 12 months: *p* = 0.010). Thus, consistently across time points, the group with severely impaired cognition was characterized by more severe negative symptoms compared to the other clusters at baseline, 6- and 12-month follow-up.

**Fig. 4.**
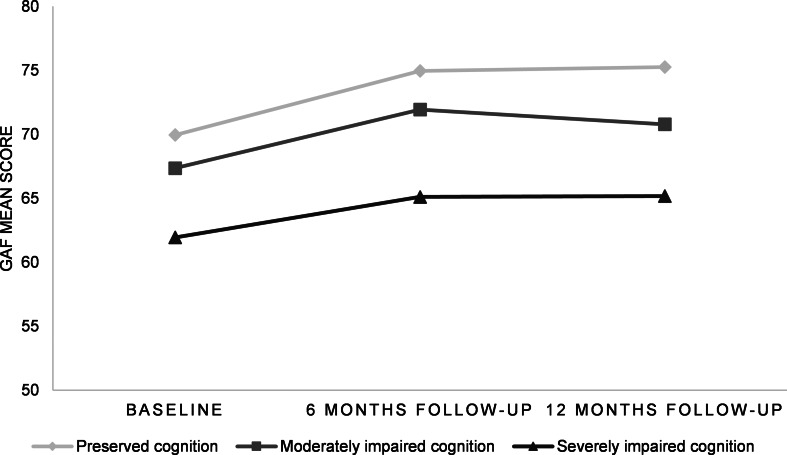
GAF mean scores illustrated for FEP cognitive clusters at baseline, 6-month follow-up and 12-month follow-up GAF, Global Assessment of Functioning; FEP, first-episode psychosis.

Furthermore, the patient subgroup with severely impaired cognition had lower clinical global functioning (total GAF score, Fig. 4) compared to patients with relatively preserved cognition, at baseline (*p* = 0.001), and trend-level effects were shown for 6-month follow-up (*n* = 144; *p* = 0.094) and 12-month follow-up (n = 132; *p* = 0.052), corrected for global functioning at baseline. In addition, lower clinical global functioning was shown in the subgroup with severely impaired cognition compared to the moderately impaired cluster at baseline (*p* = 0.045) and 6-month follow-up (*p* = 0.047).

### Functional outcome

Self-reported global functioning and disability were evaluated by the WHODAS 2.0. Although the clusters did not significantly differ across all time points, corrected for global functioning and disability at baseline (all *p* > 0.05), there was a gradual and stepwise increase in disability, with the relatively preserved cluster having lower disability scores compared to the moderately impaired and severely impaired cluster.

## Discussion

To the best of our knowledge, this is the largest study investigating cognitive subgroups of FEP patients who all reached symptomatic remission after treatment in relation to longitudinal clinical and functional outcomes. We found three distinct cognitive subgroups in a sample of FEP, including one relatively large subgroup with preserved cognition (37.2%), one moderately impaired group (36.3%) and one severely impaired group (26.5%) as compared to healthy controls. Of note, the severely impaired group included only one-fourth of the sample. The cognitive subgroups were characterized by significant differences in clinical symptoms, with more severe clinical symptoms in the severely impaired cognitive cluster compared to the relatively preserved cluster, at baseline (PANSS total and all subscales) and 6- and 12-month follow-up (PANSS negative subscale). In addition, evaluation of global functioning (GAF) was significantly higher in the relatively preserved cluster compared to the severely impaired cluster at baseline and showed trend-level effects at 6- and 12-month follow-up. No significant differences in self-reported measures of functional outcome (WHODAS 2.0) were found between the patient subgroups at baseline and follow-up, yet the same trend could be observed.

The current results provide support for cognitive heterogeneity in FEP, delineated by three cognitive subtypes. This is consistent with previous clustering studies reporting on three subgroups of cognition in both first episode and chronic samples of schizophrenia (Carruthers *et al*. 2019; Gilbert *et al*. 2014; Menkes, Armstrong, Blackford, Heckers, & Woodward, 2019; Sauvé, Malla, Joober, Brodeur, & Lepage, 2018; Uren *et al*. 2017; Wells *et al*. 2015). The relatively preserved subgroup did not perform worse on overall cognition compared to the healthy controls, confirming the existence of a subset of patients with relatively intact cognitive performance (Ammari *et al*. 2014; Carruthers *et al*. 2019; Menkes *et al*. 2019; Moritz *et al*. 2017; Uren *et al*. 2017). Although cognitive impairment has long been recognized as a core symptom of psychotic disorders, our results show that a significant proportion of patients (37.8%) perform in the same range as healthy controls. This underscores the importance of taking individual variability into account in both research and clinical practice. It should be noted that cognitive performance similar to that of healthy controls is not necessarily synonymous with cognitively unaffected. However, no differences in years of education were observed between the relatively intact subgroup and healthy controls, and no decline relative to parents' years of education was observed, suggesting that cognitive functioning did not decline relative to a higher premorbid level (Keefe, Eesley, & Poe, 2005). We further showed that both the moderately and severely impaired subgroups had attained significantly fewer years of education compared to the relatively preserved subgroup. The moderately impaired subgroup showed global cognitive impairment compared to the healthy controls, including all subdomains except for executive function. Findings regarding the intermediate cluster show global impairments of cognitive performance rather than domain-specific deficits. This is in line with previous studies performed in both first episode and chronic schizophrenia samples, which identified an intermediate cluster with overall moderate cognitive impairment (Lewandowski, Sperry, Cohen, & Öngür, 2014; Uren *et al*. 2017; Van Rheenen *et al*. 2016). The severely impaired subgroup (25.5%) showed pronounced cognitive impairments that were not restricted to specific domains, with more severe performance deficits compared to the other cognitive subgroups. The existence of a severely impaired cognitive subgroup has been previously demonstrated (Lewandowski *et al*. 2014; Uren *et al*. 2017; Van Rheenen *et al*. 2016). However, the percentage of individuals showing severely impaired cognition in this study is lower than the 44% reported in a large recent systematic review (Carruthers *et al*. 2019). Remarkably, executive function was relatively spared across all subgroups of cognition, although previous FEP studies demonstrated reduced executive function compared to healthy controls (Kravariti *et al*. 2009). Attention and speed of processing showed most severe impairments across all subgroups, which is in line with previous studies performed in FEP (Kravariti *et al*. 2009; Leeson *et al*. 2010; Weinberg *et al*. 2016)

Our finding of more severe clinical symptoms, specifically negative symptoms in the group with severely impaired cognition is in line with previous research demonstrating an association between cognitive function and negative symptoms in both FEP (Engen *et al*. 2019; Reser, Allott, Killackey, Farhall, & Cotton, 2015; Uren *et al*. 2017) and chronic schizophrenia (Lewandowski *et al*. 2014; Weinberg *et al*. 2016; Wells *et al*. 2015). However, the relationship between cognitive function and negative symptoms seems complex. Severe negative symptoms such as lack of motivation or decreased effort may impact cognitive performance but similarly, cognitive impairment could affect the manifestation of negative symptoms as more preserved cognitive function may be essential for the ability to plan, initiate, motivate and carry out daily activities (Beck, Himelstein, Bredemeier, Silverstein, & Grant, 2018; Fervaha *et al*. 2014; Fortgang, Srihari, & Cannon, 2020; Jurado & Rosselli, 2007; Lindgren *et al*. 2020). More longitudinal studies are required to gain more insight into the relationship between cognitive function and negative symptoms in FEP.

In the subgroup of individuals with severely impaired cognition, we found lower objectively evaluated global functioning (GAF) when compared to the relatively preserved subgroup. These findings are substantiated by other studies suggesting that global functioning is related to cognitive cluster membership (Gilbert *et al*. 2014; Lewandowski *et al*. 2014; Uren *et al*. 2017; Wells *et al*. 2015). Moreover, studies investigating cognitive subtypes in both psychotic patients and unaffected siblings showed that patients with cognitively impaired siblings reflect a poorer course of the disease. This suggests that cognitive impairment may indeed be predictive for the course of illness (Burger *et al*. 2021; Quee *et al*. 2014). However, no significant differences between cognitive subgroups could be demonstrated on self-reported measures of functional outcome (WHODAS 2.0). This is remarkable, as both the GAF and the WHODAS 2.0 assess measures of outcome. It is plausible that not all types of cognition are associated with the evaluation of functional outcomes. It has been suggested that not global cognition but specifically social cognition plays a critical role in outcome regarding everyday functioning. A recent study by Kim *et al*. (2021) demonstrated significant correlations between the WHODAS 2.0 and social cognition, such as communication and learning abilities (Kim *et al*. 2021). Similarly, Tan, Rossell, and Lee (2020) demonstrated that mostly verbal-linguistic cognitive skills such as semantics and language are associated with subjective measures of functioning and wellbeing, as those have a direct effect on community functioning (Tan *et al*. 2020). Indeed, medium to large associations between social cognition and community functioning have been reported in a meta-analysis (Fett *et al*. 2011), whereas only small to moderate associations have been reported between nonsocial cognition and functional outcome (Halverson *et al*. 2019). This suggests that interventions targeting social cognition may improve functional outcomes more than neurocognitive interventions. Another explanation for the lack of differences in WHODAS 2.0 between the cognitive subgroups may be the lack of awareness of functioning and disability in patients as the accuracy of assessing daily functioning in patients with schizophrenia is under debate (Jongs *et al*. 2020). An overestimation of functioning by the patient may be affected by disease-related factors such as negative symptomatology and lack of insight (Jongs *et al*. 2020; Sabbag *et al*. 2012). This indicates that despite symptoms or restrictions in clinician observed functioning, patients may be satisfied with their lives and consider their level of functioning high. Thus, our findings suggest that daily functioning from a patient's perspective is not necessarily synonymous with the clinician's interpretation of recovery and may be related to a different set of predictors. Finally, the WHODAS 2.0 includes domains of daily functioning that are hardly affected in the current FEP sample and only minimally associated with cognitive function, such as mobility (getting around, standing up, walking a long-distance) and self-care (getting dressed, washing, eating) and hence do not differentiate between the groups (Chen *et al*. 2018).

### Strengths, limitations and future directions

A strength of the current study is its large sample size and longitudinal design, evaluating both clinical and functional outcomes over a 12-month follow-up period. The participants were included shortly after diagnosis and had all achieved symptomatic remission before the baseline measurement. Therefore, factors that may influence cognitive function, such as long-term antipsychotic medication use or duration of illness, are being limited. In addition to the assessment of clinical outcome (PANSS and GAF) by trained central raters, we extensively measured functioning and disability with the self-reported WHODAS 2.0 questionnaire, which is recommended for the assessment of functioning in the DSM-5 (Gold, 2014). We also note that our study comes with some limitations. First, antipsychotic medication use was not stable for all participants throughout follow-up as some participants may have tapered off their antipsychotic medication gradually. However, the process of medication discontinuation also occurs in the general population of first-episode patients. Furthermore, although we did not include a cumulative dose of antipsychotic medication as a factor, all participants were in a similar early stage of the illness (3 to 6 months in remission of their first psychotic episode) at the time of inclusion and we found that current chlorpromazine equivalents were not significantly different between clusters. Moreover, although cognitive performance at baseline was not affected by psychotic symptoms as solely patients in symptomatic remission were included, generalizability to wider FEP populations may be limited within this study. Finally, cluster analyses come with the limitation that the determination of the number of clusters may be arbitrary as it depends on the methods used. However, we followed the recommended guidelines for reporting on cluster analysis (Carruthers *et al*. 2019).

## Conclusion

The results of the present study provide strong support for high heterogeneity in cognition among FEP patients who reach symptomatic remission. Besides finding a moderately impaired and severely impaired subgroup, we also show that a significant subset of patients have relatively preserved cognitive function. This underscores the importance of taking individual variability into account. In addition, we found that FEP patients with severe cognitive impairment have poor clinical outcomes compared to those with relatively preserved cognitive function. These findings suggest that grouping patients in subtypes along the cognitive continuum may offer crucial information about illness profiles and clinical prognosis. In conclusion, early identification of distinct cognitive profiles in FEP and corresponding longitudinal differences in clinical profile has clear implications for prognosis and personalized treatment of psychotic disorders. However, self-reported measures of functional outcome seem to have different sets of predictors in FEP and more longitudinal studies are required to further assess determinants of functional outcome.

## Supplementary material

For supplementary material accompanying this paper visit https://doi.org/10.1017/S0033291721004153. click here to view supplementary material
