# Supplementary material for: Longitudinal clinical and functional outcome in distinct cognitive subgroups of first-episode psychosis: a cluster analysis
Source: Psychol Med. 2021 Oct 19;53(6):2317–27. doi: 10.1017/S0033291721004153 (PMC10123843; doi:10.1017/S0033291721004153)
Supplement: Supplementary file 1 [file S0033291721004153sup001.docx]

**Supplementary Material**

**Supplementary Table S1. Conversion values of educational levels.***Conversion values of the highest achieved and completed educational level*

| **Educational level (CASH-III)** | **Years of education** |  |
| --- | --- | --- |
| None | 0 |  |
| Primary school | 6 |  |
| Lower secondary education/domestic science school | 10 |  |
| Middle general secondary vocational education | 10 |  |
| Higher general secondary education | 11 |  |
| Pre-university education | 12 |  |
| Community college/intermediate vocational education | 14 |  |
| Higher vocational education | 15 |  |
| Academic education | 17 |  |
| CASH-III, Comprehensive Assessment of Symptoms and History part III Andreasen et al., 1992) | | |


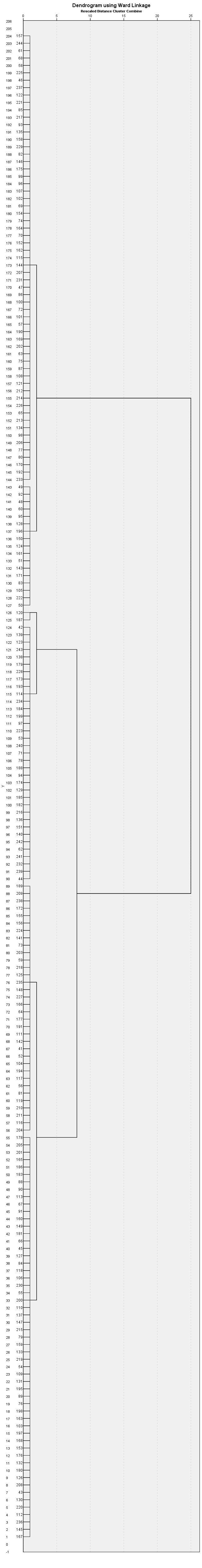
**Supplementary Figure S1. Dendrogram Hierarchical Clustering Analysis using Ward’s Method**

**Supplementary Figure S2. Agglomeration schedule coefficients Hierarchical Clustering Analysis using Ward’s Method**


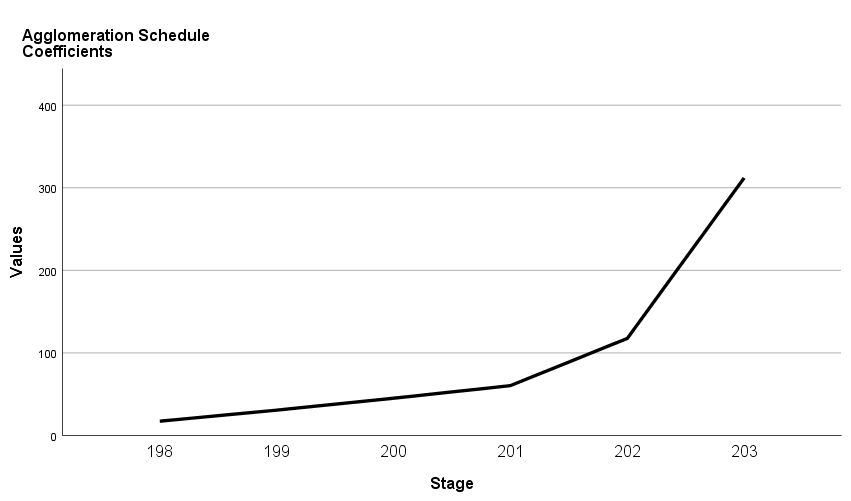


**Supplementary Table S2. Mean (SD) baseline demographic, cognitive and clinical characteristics for FEP patients and healthy controls**

|  |  |  |  |  |  |
| --- | --- | --- | --- | --- | --- |
|  | FEP patients (n=204) | Healthy controls (n=40) | Test statistic  F, χ2 | df | p-value |
| Male, n (%) | 148 (72.5%) | 32 (80.0%) | χ2 = 0.96 | 1 | p = 0.327 |
| Age | 27.93 (8.90) | 24.48 (4.98) | F = 5.67 | 1 | p = 0.018 |
| Years of education | 13.77 (2.53) | 14.95 (1.95) | F = 7.71 | 1 | p = 0.006 |
| Years of education parents | 12.84 (3.30) | 13.63 (2.20) | F = 2.06 | 1 | p = 0.153 |
| Chlorpromazine equivalent | 238.67 (137.68) | N.A. | N.A. | N.A. | N.A. |
| **BACS, Z-score** |  |  |  |  |  |
| Composite score | -1.44 (1.24) | 0.13 (1.15) | F = 54.34 | 1 | p < 0.001 |
| Verbal memory | -0.70 (1.08) | 0.43 (1.01) | F = 37.29 | 1 | p < 0.001 |
| Working memory | -0.95 (1.20) | 0.07 (1.05) | F = 25.23 | 1 | p < 0.001 |
| Motor speed | -0.95 (1.37) | -0.15 (0.96) | F = 12.17 | 1 | p = 0.001 |
| Verbal fluency | -1.06 (1.16) | 0.11 (1.05) | F = 35.05 | 1 | p < 0.001 |
| Attention & Processing speed | -1.32 (0.90) | -0.23 (1.19) | F = 42.77 | 1 | p < 0.001 |
| Executive function | -0.17 (1.25) | 0.24 (0.87) | F = 3.98 | 1 | p = 0.047 |
| **PANSS** |  |  |  |  |  |
| Total | 44.24 (10.06) | N.A. | N.A. | N.A. | N.A. |
| Positive | 9.34 (2.74) | N.A. | N.A. | N.A. | N.A. |
| Negative | 11.84 (4.32) | N.A. | N.A. | N.A. | N.A. |
| General | 23.05 (5.22) | N.A. | N.A. | N.A. | N.A. |
| FEP=first episode psychosis; PANSS=Positive and Negative Syndrome Scale; BACS=Brief Assessment of Cognition in Schizophrenia; | | | | | |
